# *Mycobacterium bovis* naturally infected calves present a higher bacterial load and proinflammatory response than adult cattle

**DOI:** 10.3389/fvets.2023.1105716

**Published:** 2023-04-27

**Authors:** Jacobo Carrisoza-Urbina, Mario A. Bedolla-Alva, Rogelio Hernández-Pando, Constantino López-Macías, Sara Huerta-Yepez, Guillermina Baay-Guzmán, Mireya Juárez-Ramírez, José A. Gutiérrez-Pabello

**Affiliations:** ^1^Laboratorio de Investigación en Tuberculosis y Brucelosis, Facultad de Medicina Veterinaria y Zootecnia, Universidad Nacional Autónoma de México, Mexico City, Mexico; ^2^Departamento de Patología, Facultad de Medicina Veterinaria y Zootecnia, Universidad Nacional Autónoma de México, Mexico City, Mexico; ^3^Sección de Patología Experimental, Departamento de Patología, Instituto Nacional de Ciencias Médicas y Nutrición Salvador Zubiran, Mexico City, Mexico; ^4^Unidad de Investigación Médica en Inmunoquímica, UMAE Hospital de Especialidades, Centro Médico Nacional Siglo XXI. Instituto Mexicano del Seguro Social (IMSS), Mexico City, Mexico; ^5^Unidad de Investigación en Enfermedades Oncológicas, Hospital Infantil de México Federico Gómez, Mexico City, Mexico

**Keywords:** atypical granulomas, *Mycobacterium bovis*, natural-infection, calcification, bovine tuberculosis, calves immune-response, proinflammatory response

## Abstract

Granulomas are characteristic bovine tuberculosis lesions; studying this structure has improved our understanding of tuberculosis pathogenesis. However, the immune response that develops in granulomas of young cattle naturally infected with *Mycobacterium bovis* (*M. bovis*) has not been fully studied. Our previous work described an atypical pattern in granulomatous lesions of cattle younger than 4 months (calves) naturally infected previously *M. bovis* that did not correspond to the histological classification previously proposed. Histologically, granulomas from calves lack a connective tissue capsule and have fewer multinucleated giant cells (MGCs) and more acid-fast bacilli (AFB) than the classic tuberculosis lesions found in cattle older than 1 year (adults); this suggests a deficient immune response against *M. bovis* infection in young animals. Therefore, we used IHC and digital pathology analysis to characterize the *in situ* immune response of granulomas from young and adult cattle. The immunolabeling quantification showed that granulomas from calves had more mycobacteria, CD3^+^ cells, IFN-γ, TNF-α, and inducible nitric oxide synthase (iNOS) than those of adult cattle. Furthermore, calf granulomas showed lower immunolabeling of MAC387^+^, CD79^+^, and WC1^+^ cells without connective tissue surrounding the lesion and were associated with less vimentin, Alpha Smooth Muscle Actin (α-SMA), and TGF-β compared with granulomas from adult cattle. Our results suggest that the immune responses in granulomas of cattle naturally infected with *M. bovis* may be age dependent. This implies that an exacerbated proinflammatory response may be associated with active tuberculosis, producing more necrosis and a lower microbicidal capacity in the granulomas of calves naturally infected with *M. bovis*.

## 1. Introduction

Bovine tuberculosis caused by *Mycobacterium bovis* affects different mammals, including humans. In the livestock industry, *M. bovis* causes losses of approximately 3 billion dollars per year (1, 2). This disease mainly affects cattle’s lymph nodes and lungs, where granulomas are formed. These structures isolate and control mycobacteria and restrict tissue damage by preventing chronic inflammation of the surrounding tissue (3). Granuloma formation depends on the recruitment and activation of the cellular immune response and the persistence of the mycobacterial antigenic stimulus. These factors will determine the histology and development of the lesion (4). The structure of the granuloma has been associated with the progression or control of the disease; granulomas with little necrosis and well delimited by cellular and connective tissue capsules are associated with a lower bacillus number than inadequately formed granulomas with extensive necrosis and limited encapsulation. The latter type of lesion has been reported in humans and experimental monkeys with active tuberculosis as well as in individuals co-infected by *Mycobacterium tuberculosis/*HIV (5–7).

We have previously characterized granulomas of cattle naturally infected with *M. bovis* and older than 1 year, observing lesions comparable to those reported by Wangoo et al. (8). In contrast, bovines younger than 4 months presented “atypical” granulomas with many bacilli, necrosis, and absence of a connective tissue capsule, suggesting that this group of calves developed a response that was unable to form a granuloma to control the infection (9). Nonetheless, the immune response present in the granulomas of these animals is unknown. To better understand the immune response of granulomas induced by *M. bovis* at the cellular and molecular levels, we used immunohistochemistry (IHC) and digital pathology analysis to characterize granulomas from cattle older than 1 year and calves younger than 4 months. Our results suggest that calf granulomas present more mycobacteria, a greater proinflammatory response, and a lack of connective tissue capsule compared to the granulomas from adults.

## 2. Materials and methods

### 2.1. Sample collection

Mediastinal lymph node samples were collected from 25 naturally infected cattle, 15 were adult Holstein-Friesian dairy cows between one to 5 years of age, and 10 corresponded to calves between 1 week to 4 months of age. These tissues were collected with owner consent, from cattle that exhibited lesions suggestive of tuberculosis in the post-mortem examination. All cattle died from conditions that did not include tuberculosis, the main circumstances of death (euthanasia, emergency slaughter or unassisted death), were metabolic/digestive disorders, pneumonias, traumatism, and mastitis/udder problems, in the Supplementary Table 1 we described the causes of death of each animal. The samples were collected in a dairy basin from the central region of Mexico with a prevalence of bovine tuberculosis higher than 16% (10).

### 2.2. Histopathological analyses of paraffin-embedded tissues

Samples of lymph nodes, lung tissue, and individual organs that exhibit tuberculosis-suggestive lesions were collected during the necropsy. Tissues were divided for histopathology and bacteriological cultures.

For histopathological analysis, the tissue was fixed in 10% formaldehyde and embedded in paraffin. From the formalin-fixed paraffin-embedded tissues (FFPE) were obtained 4-μm width serial sections and stained with Hematoxylin and Eosin (H&E), Masson’s trichrome, Ziehl Neelsen (ZN), and Von Kossa. In these sections were identified granulomas with fibrous tissue capsules, acid-fast bacilli, and calcification. Granulomas were identified and staged according to Wangoo et al. (8) and using a new classification of granulomas in the group of young bovines (9).

### 2.3. *Mycobacterium bovis* identification

*Mycobacterium bovis* was identified by bacteriological isolation and by PCR of FFPE tissues. Briefly, part of the collected tissue was used for bacteriological isolation after Petroff’s decontamination method under biosecurity conditions (11). To confirm the presence of *M. bovis,* we extracted genomic DNA from the bacteriological isolation and FFPE tissues that had granulomas. In the case of paraffin blocks, 10 to12 micron sections were obtained by use of a microtome and added to a 1.5 ml centrifuge tube, microtome blades were cleaned with 70% alcohol between slices to avoid cross-contamination of samples. After, 1 ml of xylol was added to each tissue section which was then vortexed and incubated for 5 min. Xylol was subsequently decanted and tissue section was washed twice with absolute ethyl alcohol, allowed to dry, and resuspended in 400 μl of TE with 50 μl of lysozyme (10 mg/ml) and were incubated over-night at 37°C. Then, we used the CTAB (N-cetyl-N, N, N-trimetyl ammonium bromide)/chloroform-isoamyl alcohol protocol, described by Van Helden et al. (12). Next, a nested PCR was performed to amplify the mpb70/m22 genes and identify members of the Mycobacterium tuberculosis complex. We used a commercial kit (TopTaq Master Mix Kit) followed the manufacture instructions. Primers for the mpb70 gene that amplify a product of 372 bp were: mpb70 F (5′-GAACAATCCGGAGTTGACAA-3′) and mpb70 R (5′-AGCACGCTGTCAATCATGTA-3′). For a second reaction a 208 bp product from the same gene was obtained, the M22 F (5′-GCTGACGGCTGCACTGTCGGGC-3′) and M22 R (5′-CGTTGGCCGGGCTGG TTTGGCC-3′) primers were used. Finally, PCR of the RD9 and RD4 genes was used to identify specifically *M. bovis.* For the RD9 gene, selected primers were RD9 F (GTGTAGGTCAGCCCCATCC), RD9 I (CAATGTTTGTTGCGCTGC) and RD9 R (GCTACCCTCGACCAAGTGTT), with a product of 333 bp for *M. tuberculosis* and 206 bp for *M. bovis* and for RD4 gene the primers were RDF (ATGTGCGAGCTGAGCGATG), RD4 I (TGTACTATGCTGACCCATGCG) and RD4 R (AAAGGAGCACCATCGTCCAC), with a product of 268 bp for *M. bovis* and *M. bovis* BCG, for the rest of the members of the *M. tuberculosis* complex a product of 172 bp is amplified (12–14).

### 2.4. Immunohistochemistry

IHC procedures are summarized in Table 1. Briefly, FFPE tissues were cut into 4–5 μm sections and placed on electrocharged slides (Kling-On Slides-Biocare Medical). The sections were deparaffinized at 60°C for 30 min, rehydrated, and placed in 3% hydrogen peroxide for 15 min to eliminate endogenous peroxidase activity; then, epitope demasking was performed using both physical and chemical methods according to the primary antibody (Table 1). The tissues were washed with distilled water and placed in Sequenza cover plates (Shandon Scientific Loughborough, UK) for immunolabeling. The sections were washed after each step of the staining procedure with phosphate-buffered saline (PBS: 138 mM NaCl, 3 mM KCl, 8.1 mM Na2HPO4, 1.5 mM KH2HPO4 adjusting the pH to 7.4) and Tris-buffered saline with Tween (TBST: 0.005 mM Tris-buffered saline, pH 7.6 with 0.05% Tween 20). A universal blocking reagent (Background Sniper BS966L10) for reducing nonspecific background staining was added, then samples were incubated with the primary antibody. Antibody concentration and incubation time were standardized for each antibody. After washing twice, MACH 1 Universal HRP-Polymer Detection (Micro-polymer detection) was used following the manufacturer’s instructions. A probe (mouse antibodies only) was added to the sections and incubated for 15 min at room temperature. Then, the Polymer was added and incubated for 30 min at room temperature, followed by 3,3′-diaminobenzidine tetrahydrochloride (DAB) for visualization. The slides were rinsed in purified water, counterstained in Mayer’s hematoxylin, dehydrated, and mounted with resin.

**Table 1:** Immunohistochemical reagents and technical procedures.

Antibody and dilution	Antibody type	Supplier	Primary antibody incubation	Epitope demasking	Buffer
MAC387 1/500	Mouse monoclonal IgG1	Bio-rad MAC387	O/N 4°C	Proteinase K	PBS
CD79 1/50	Mouse monoclonal IgG1	Dako HM57	O/N 4°C	Proteinase K	PBS
CD3 1/50	Rabbit polyclonal IgG	Biocare medical SP7	O/N 4°C	Citric acid buffer, pH 6.0	TBST
Vimentin 1/100	Rabbit polyclonal IgG	Biocare medical CRM 312	45 min	Citric acid buffer, pH 6.0	TBST
Smooth Muscle Actin 1/100	Mouse monoclonal IgG1	Biocare medical SP9	45 min	Citric acid buffer, pH 6.0	TBST
Anti-*Mycobacterium* 1/100	Rabbit polyclonal	Biocare medical CP 140	O/N 4°C	Citric acid buffer, pH 6.0	TBST
TGF-β 1/100	Mouse monoclonal IgG1	Gene Tex TB21	O/N 4°C	Citric acid buffer, pH 6.0	TBST
TNF-α 1/100	Mouse monoclonal IgG1	Gene Tex CC327	O/N 4°C	Citric acid buffer, pH 6.0	TBST
iNOS 1/500	Rabbit polyclonal	Millepore 06–573	45 min	Citric acid buffer, pH 6.0	TBST
IFN-γ 1/100	Mouse monoclonal IgG1	Gene tex CC330	O/N 4°C	Citric acid buffer, pH 6.0	TBST
WC1 1/500	Mouse monoclonal IgG1	Invitrogen, CC15	O/N 4°C	Citric acid buffer, pH 6.0	TBST

O/N = 18–20 h in a refrigerator (+4°C).

TBST = 0.005 mM Tris-buffered saline, pH 7.6 with 0.05% Tween 20.

PBS: 138 mM NaCl, 3 mM KCl, 8.1 mM Na_2_HPO_4_, and 1.5 mM KH_2_HPO_4_, pH 7.4.

### 2.5. Digital image analysis in granulomas

The slides immunolabeled with different antibodies were processed digitally with a scanning microscope (Aperio Scanscope CS, Aperio, CA, USA), generating 40× images with a spatial resolution of 0.45 μm/pixel. Images were analyzed with the ImageScope software (Aperio, CA, USA), and granulomas were delimited by removing the areas of necrosis composed of cell debris and calcification. Various algorithms were used to standardize the adequate detection level for the quantification of the brown staining obtained from IHC. This methodology enabled the quantification of the proteins marked in the different IHC tests. Supplementary Figure 1 summarizes the experimental procedures used in this study, and Supplementary Table 2 shows the antibodies used and the number of granulomas analyzed in each group.

### 2.6. Statistical analyses

Statistical analysis was performed using the PASW Statistics 18 program and GraphPad Prism 7.0. Shapiro–Wilk test and normal Q-Q plots were tested for normality. Comparisons of immunostaining in granuloma between adult and young cattle were performed by the nonparametric Mann–Whitney test. Significant differences were considered when *p* < 0.05.

## 3. Results

### 3.1. Granulomas of calves naturally infected by *Mycobacterium bovis* exhibit high numbers of bacteria

**Figure 1:**
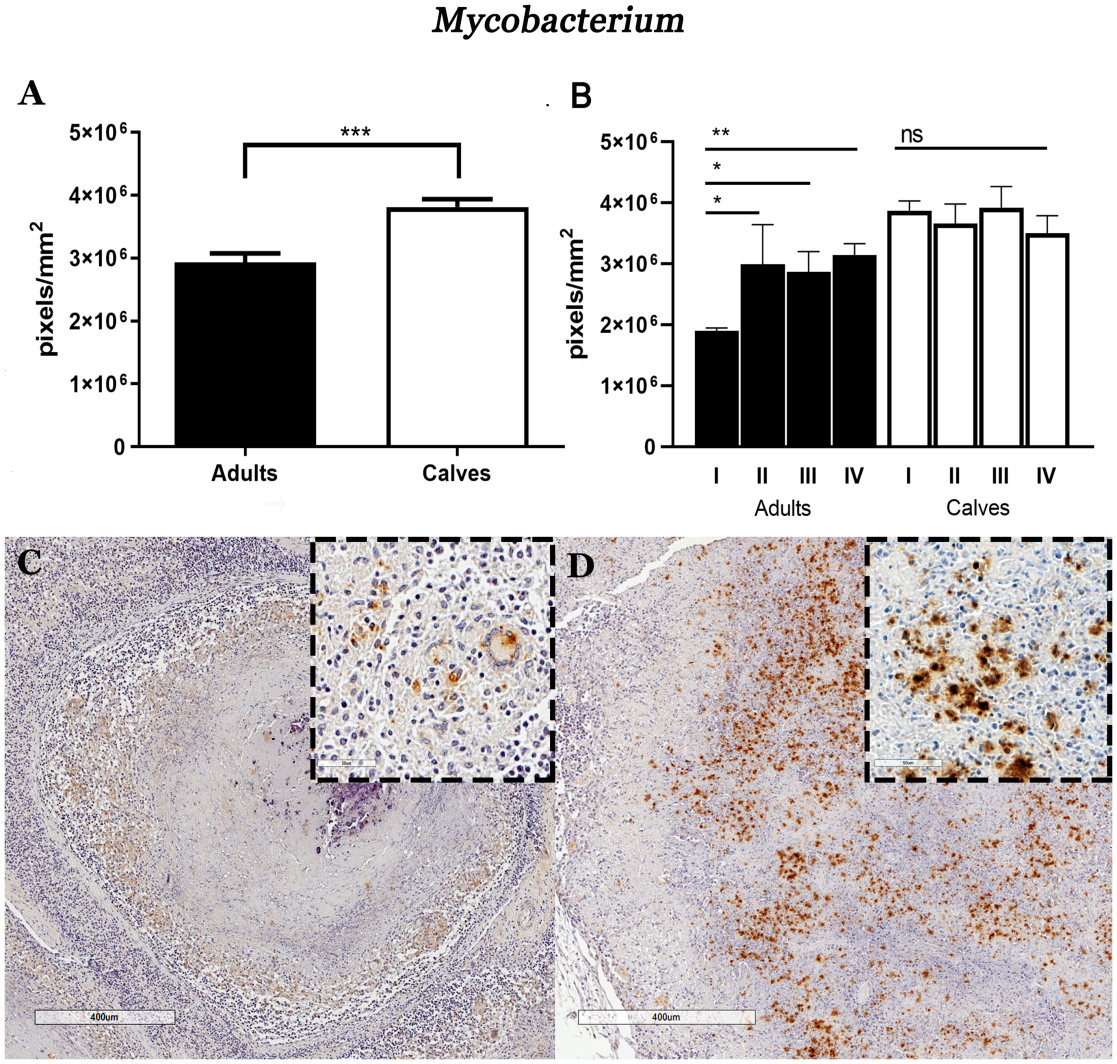
Granulomas of calves *Mycobacterium* *bovis* naturally infected show higher mycobacteria immunolabeling compared to adult cattle. **(A)** Average number of pixels positive for the IHC anti-mycobacterium staining in granulomas and stages from adult and young cattle, respectively. **(B)** Mann–Whitney test *p < 0.05, **p < 0.01, and ***p < 0.001. **(C**,**D)** Microphotograph of IHC anti-mycobacterium, 40×. **(C)** Stage IV adult bovine granuloma with faint positive brown areas around the necrosis, close-up showing cells with macrophagic morphology and a giant cell with a cytoplasmic mark. Granuloma from calf showing a large amount of positive staining in the necrotic area: the enclosure shows cytoplasmic and extracellular immunolabeling.

Formalin-fixed paraffin-embedded lymph node sections from 25 Holstein Friesian cattle naturally infected with *M. bovis* were used. IHCs were performed on these tissues to identify cell populations, cytokines, and the presence of mycobacteria. A total of 3,439 granulomas were analyzed, of which 31.3% (1,077) were from adult cattle, and 68.6% (2,362) were from calves. Using the Ziehl–Neelsen staining, we had previously observed more AFB in granulomas of the mediastinal lymph nodes of calves compared with adult cattle. To validate this result and quantify the number of bacteria, we performed IHC using a polyclonal anti-mycobacterium antibody; we confirmed higher immunostaining in granulomas from calves than in those from adult cows as well stage comparison presented the same pattern (Figures 1A,B; Supplementary Figure 1). The higher sensitivity of this technique allowed us to detect not only the presence of bacilli, but also cellular remains in the forms of vacuoles and cytoplasmic dust that are possibly associated with cell debris due to mycobacterial processing and phagocytosis. This staining was observed mainly in the cytoplasm of macrophages (MΦs), epithelioid MΦs, and multinucleated giant cells (MGCs). Positive staining was scarcely found in granulomas from adult cattle, which presented necrotic centers with mineralization and were circumscribed by a connective tissue capsule; in these types of lesions, the positive immunostaining was only observed in the MGCs (Figure 1C). In calf granulomas, positive staining was extracellular and predominant in necrotic areas (Figure 1D). Interestingly, the cytoplasm of different types of cells outside the granulomas had cytoplasmic dust immunolabeling in both groups. These results suggested that the higher bacteria burden found in granulomas of calves compared with adult cattle may be correlated with the type of immune response. Therefore, we sought to identify the major cell populations and cytokines associated with the immunopathology of tuberculosis.

### 3.2. Calf granulomas do not develop a fibrous capsule

**Figure 2:**
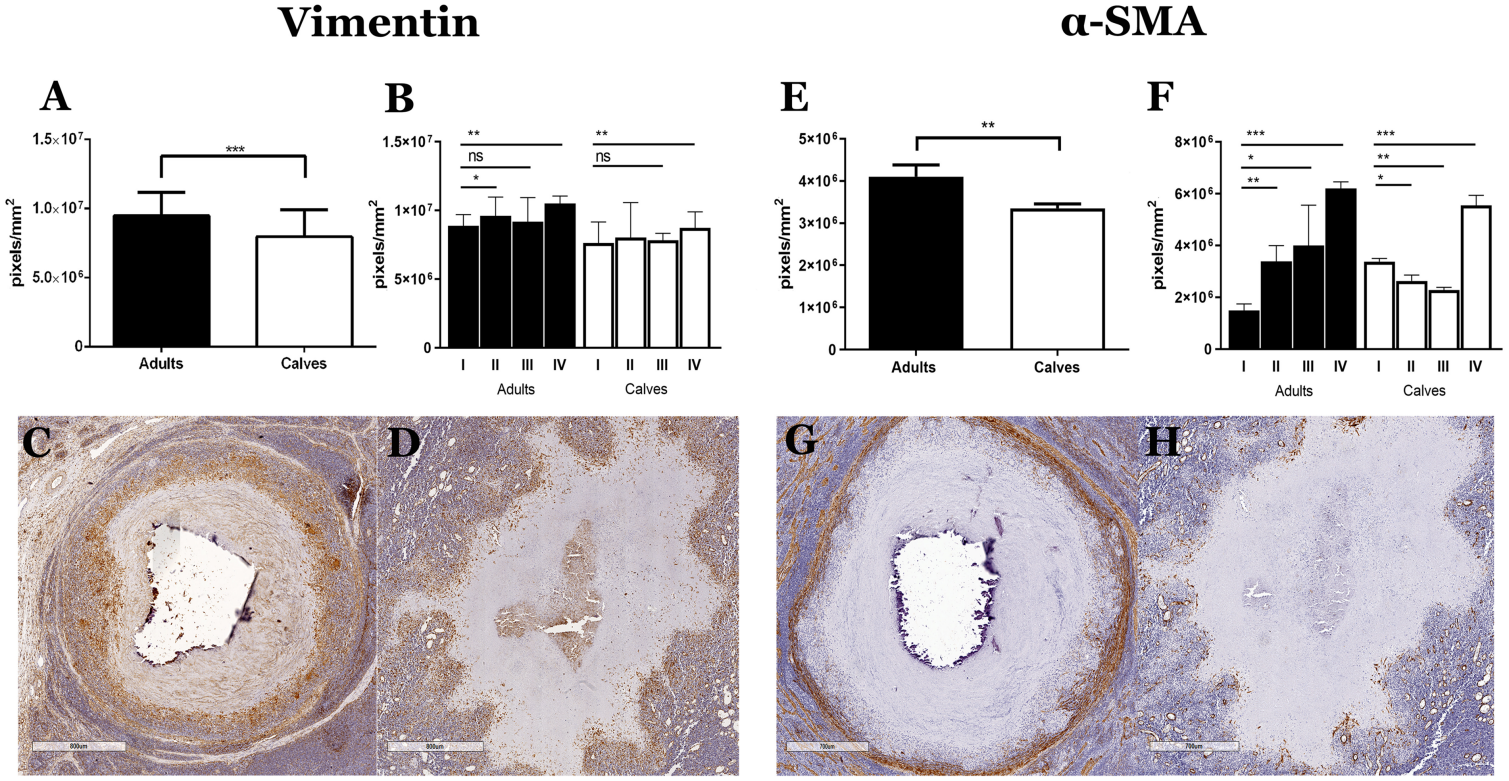
Absence of connective tissue capsule in granulomas of calves naturally infected by *Mycobacterium bovis* is associated with a lower number of fibroblasts and myofibroblasts. **(A,B** and **E,F)** Expression of vimentin and α-SMA immunolabeling was quantified in the granulomas and stages of adults and calves, Mann–Whitney test *p < 0.05, **p < 0.01, and ***p < 0.001, respectively. **(C,D)** IHCs of vimentin in late granulomas show abundant staining mainly in the connective tissue capsule of granuloma from adult cattle compare with calf granuloma, respectively. **(G,H)** IHCs of α-SMA in late granulomas show myofibroblast forming the connective tissue capsule in granuloma from adult cattle. However, granuloma from a calf showing mixed positive cells around the necrosis without forming a connective tissue capsule.

One of the main findings of the histopathological analysis in the tissue stained with Masson’s trichrome was the absence of a connective tissue capsule in the granulomas of calves, even in the presence of necrosis and calcification. Fibroblasts and myofibroblasts have been reported as the main cell populations that form the connective tissue capsules in the granulomas caused by *M. bovis*; these capsules are mainly composed of type I collagen (8). We observed greater vimentin (fibroblasts) and α-SMA (myofibroblast) immunolabeling in adult cattle compared with calves (Figure 2). Vimentin staining was identified in epithelioid MΦs, MGCs, and mostly in cells with fibroblast characteristics, which were interspersed in the cellular area of the granulomas. Positive vimentin fibroblasts formed cell layers with different thicknesses comprising the capsule of connective tissue around granulomas in stages III and IV of adult cattle; this distribution of fibroblasts around the lesions was absent in calf granulomas (Figures 2C,D). Immunostaining of α-SMA was found in cells with fibroblast morphology. Initial granulomas from both groups showed positive α-SMA interspersed with epithelioid MΦs and lymphocytes, showing more positive cells in calf granulomas stage I (Figures 2E,F; Supplementary Figure 3). In adult cattle granulomas of stages III and IV, α-SMA positive cells were embedded in the connective tissue capsule, and the thickness of the capsule was related to the number of the myofibroblast layers. In late granulomas from calves, the positive cells were irregularly distributed and interspersed with the other cells without forming a capsule around the lesion (Figures 2G,H).

### 3.3. *Mycobacterium bovis* granulomas from calves and adult cattle have different cell proportions

**Figure 3:**
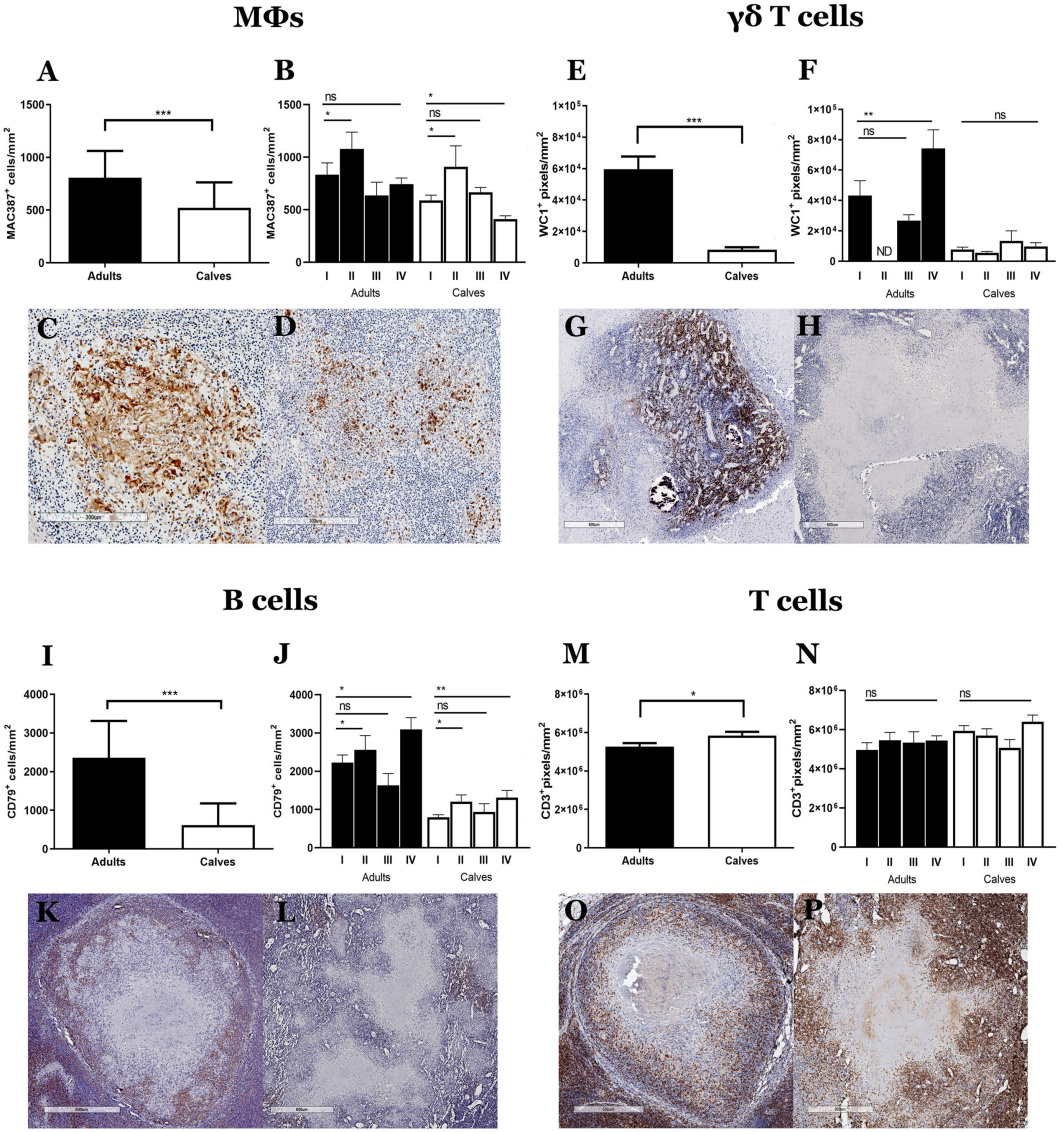
Cell population differs between granulomas from calves and adult cattle naturally infected with *Mycobacterium bovis*. **(A–P)** Average expression and representative images of immunolabeling for MAC387 (macrophages), WC1 (γδ T cells), CD79 (B cells), and CD3 (T cells), respectively, in granulomas and stages from adult cattle and calves, Mann–Whitney test *p < 0.05, **p < 0.01, and ***p < 0.001. **(C)**, 100× Stage II granuloma from an adult showing abundant MAC387^+^ cells, including some MGCs. **(D)** 100× Granuloma from a calf with positive cells around a necrotic area. **(G)**, 40× Stage IV granuloma from an adult with abundant staining of WC1+cells (γδ T cells) in the center of the lesion. **(H)**, 40× Granuloma from a calf with a few WC1+cells. **(K)**, 40× CD79^+^ cells around the granulomas; stage IV granuloma staining around the lesion. **(L)**, 40× Granuloma from a calf, with some CD79^+^ cell aggregates around the necrotic area. **(O,P)**, 40× Intense staining of CD3^+^ cells around the necrosis interspersed between the rest of the cells that form the granuloma in adults and calves, respectively. ND, not determined (because of lack to adult granulomas stage II).

Granulomas from calves have more bacteria, no connective tissue capsules, and a lower number of fibroblasts and myofibroblasts than those from adult cattle. Taken together, these results suggest a functional difference in the immune response that is probably related to the type of cells that form the granulomas. We used IHC to identify the main cell populations of granulomas from adult cattle and calves. Granulomas from adult cattle showed more MAC387 (MΦs/monocytes), WC1 (γδ T cells), CD79^+^ (B lymphocytes) cells, and fewer CD3^+^ (T lymphocytes) cells compared to granulomas from calves In addition, we evaluated the differences between stages of granulomas, and we observed the same pattern (Figure 3; Supplementary Figure 3). Adult’s granulomas showed more MΦs, epithelioid MΦs, and MGCs than calves’ granulomas (Figures 3A,D). γδ T^+^ cells were present in granulomas, as well as in the parenchyma of the lymph nodes. An increasing number of positive cells correlated with the granuloma stage in adult cattle; stages III-IV showed many positive cells in the cellular area and around the connective tissue capsule in adult’s granulomas, whereas few γδ T^+^ cells were observed in all granuloma stages from calves (Figures 3E,H). Adult’s granulomas showed more B lymphocytes located between the cellular area of the initial stages and around the lesions with calcification and necrosis that calves´ granulomas. Interestingly, B cells niches were found in some late granulomas in both groups (Figures 3I,L). Finally, T lymphocytes were observed in both groups surrounding the tissue capsule; they were interspersed in the initial stages and surrounding the necrosis area in later stages. A slight increase of these cells was observed in calves compared to adult’s granulomas (Figures 3M,P).

### 3.4. A higher proinflammatory response was observed in granulomas from calves compared to adults

**Figure 4:**
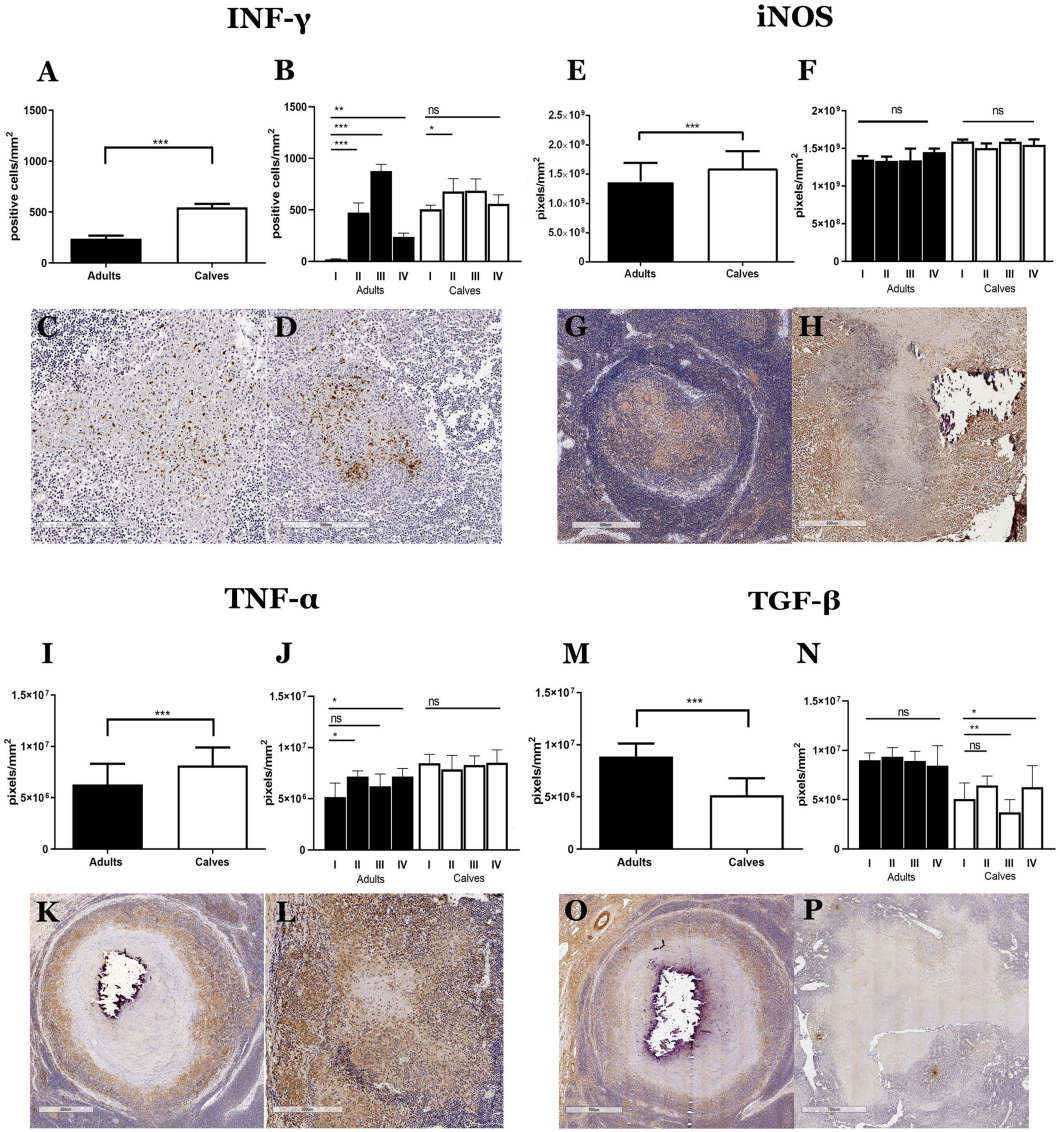
Granulomas from calves show a more proinflammatory response than adults. **(A–P)** Average expression and representative images of immunolabeling for IFN-γ, the inducible form of nitric oxide synthase (iNOS), TNF-α, and TGF-β, respectively, in granulomas from adults and calves; Mann–Whitney test *p < 0.05, **p < 0.01, and ***p < 0.001. **(C,D)**, 100× Initial granulomas showing IFN-γ^+^ cells with lymphocyte morphology in the center of the lesion. **(G,H)**, 40× iNOS staining around necrosis, positive cells with macrophage morphology, and some MGCs with different staining intensities from adults and calves, respectively. **(K)**, 40×; **(L)**, 100× TNF-α^+^ cells and extracellular staining around necrosis, positive cells have an epithelioid MΦs morphology, and some MGCs show different staining intensity in adults and calves, respectively. **(O,P)**, 40× TGF-β staining is observed extracellularly and in the cytoplasm of epithelioid MΦs, some MGCs, and fibroblast cells.

We hypothesized that the differences in cell populations observed in the granulomas of calves and adult cattle are related to a distinct type of immune response. To further explore this hypothesis, we evaluated cytokines and inflammatory mediators associated with tuberculosis immunopathology in humans and cattle, such as gamma interferon (IFN-γ), an inducible form of nitric oxide synthase (iNOS), transforming growth factor-beta (TGF-β) and tumor necrosis factor α (TNF-α) (4, 15, 16). Although proinflammatory cytokines have been associated with mycobacterium infection control, we observed more immunolabeling of IFN -γ, iNOS, and TNF-α and less TGF-β in the calf granulomas compared to adult granulomas. The same pattern was observed in stages of granulomas between calves and adult cattle (Figure 4; Supplementary Figure 4). Intracellular IFN-γ staining was observed mainly in lymphocytes. In both groups, initial-stage granulomas presented IFN-γ^+^ cells mixed with epithelioid MΦs, whereas late-stage granulomas showed IFN-γ staining in areas surrounding the necrosis. Interestingly, there was a higher number of IFN-γ^+^ cells in stage III granuloma of adult cattle compared to calves (Figures 4A,D; Supplementary Figure 4A). A higher expression of iNOS was detected in granulomas and stages of calves compared to adults. The staining was generally observed in the cytoplasm of epithelioid MΦs and MGCs, although not all giant cells present in the same granuloma were positive (Figures 4E,H). TNF-α immunolabeling was greater in granulomas of calves observed in the cytoplasm of epithelioid MΦs, MGCs, and some fibroblasts found in cellular areas of granulomas and extracellularly, with greater intensity around necrotic sections and less intensity in the periphery of the lesion (Figures 4I,L). Finally, TGF-β, an anti-inflammatory cytokine, was observed in the extracellular and cytoplasmic area of epithelioid MΦs and MGCs from both groups. High TGF-β staining was observed in fibroblasts that form the connective tissue capsule in stage III-IV granulomas from adult cattle compared to calves (Figures 4M,P). All these results suggest an exacerbated proinflammatory process that causes an inability to control the infection by *M. bovis* in naturally infected cattle.

### 3.5. Concentration of INF-γ and gamma delta T cells is granuloma stage-dependent

**Figure 5:**
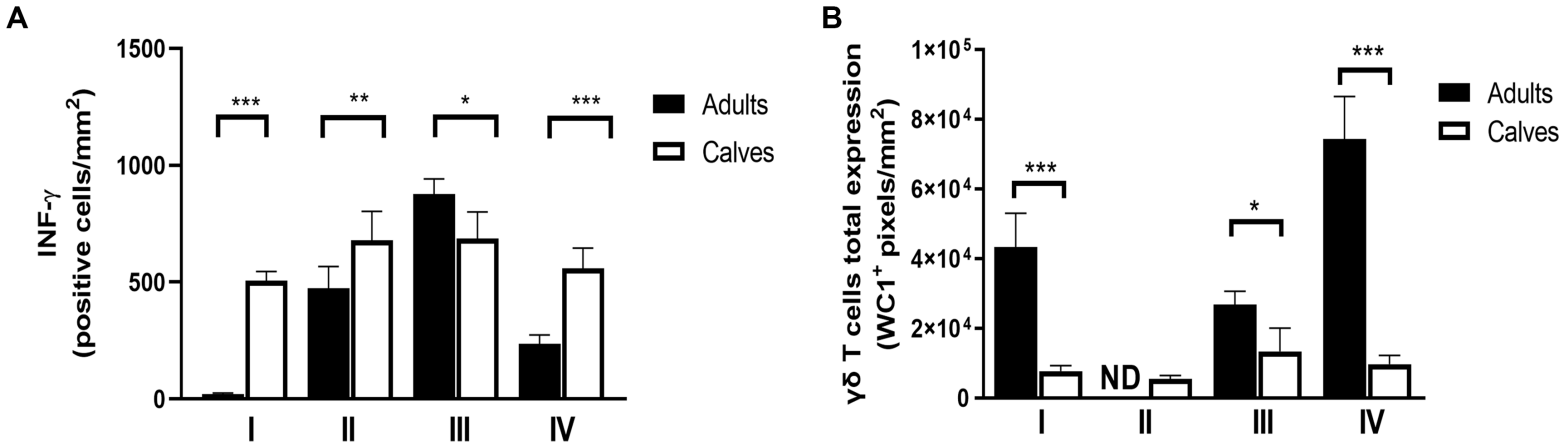
INF-γ and gamma delta T cells form granulomas have stage dependent concentration. **(A)** Average expression of immunolabeling for IFN-γ and **(B)** WC1 (γδ T cells), respectively, in granuloma stages from adult cattle and calves. Mann–Whitney test *p < 0.05, **p < 0.01, and ***p < 0.001. ND, not determined (because of lack to adult granulomas stage II).

When analyzing the granulomas by stage and did the comparison between adult and calves, a variation in the labeling of INF-γ and γδ T cells was observed. Although the global average of INF-γ is higher in granulomas of calves, in adult cattle we observed higher concentration of this cytokine in stage III granulomas. The immunolabeling of γδ T cells is higher in adults compare to calves. It is interesting to note that the highest concentration of γδ T cells is observed in stage IV granuloma. The amount of INF-Y does not correlate with the expression of γδ T cells observed in these lesions (Figures 5A,B).

## 4. Discussion

Granulomas are the characteristic lesions of bovine tuberculosis. The development, morphology, and fate of this structure depend on several factors, including chronic stimulation by the virulent mycobacteria and the host’s immune response associated with the type of cell population, cytokines, chemokines, and cell activation. The immune response and morphological characteristics of *M. bovis* granulomas have been studied mainly in cattle older than 6 months of age (4). However, very little information has been reported on the immunology of tuberculosis in young animals (17).

**Figure 6:**
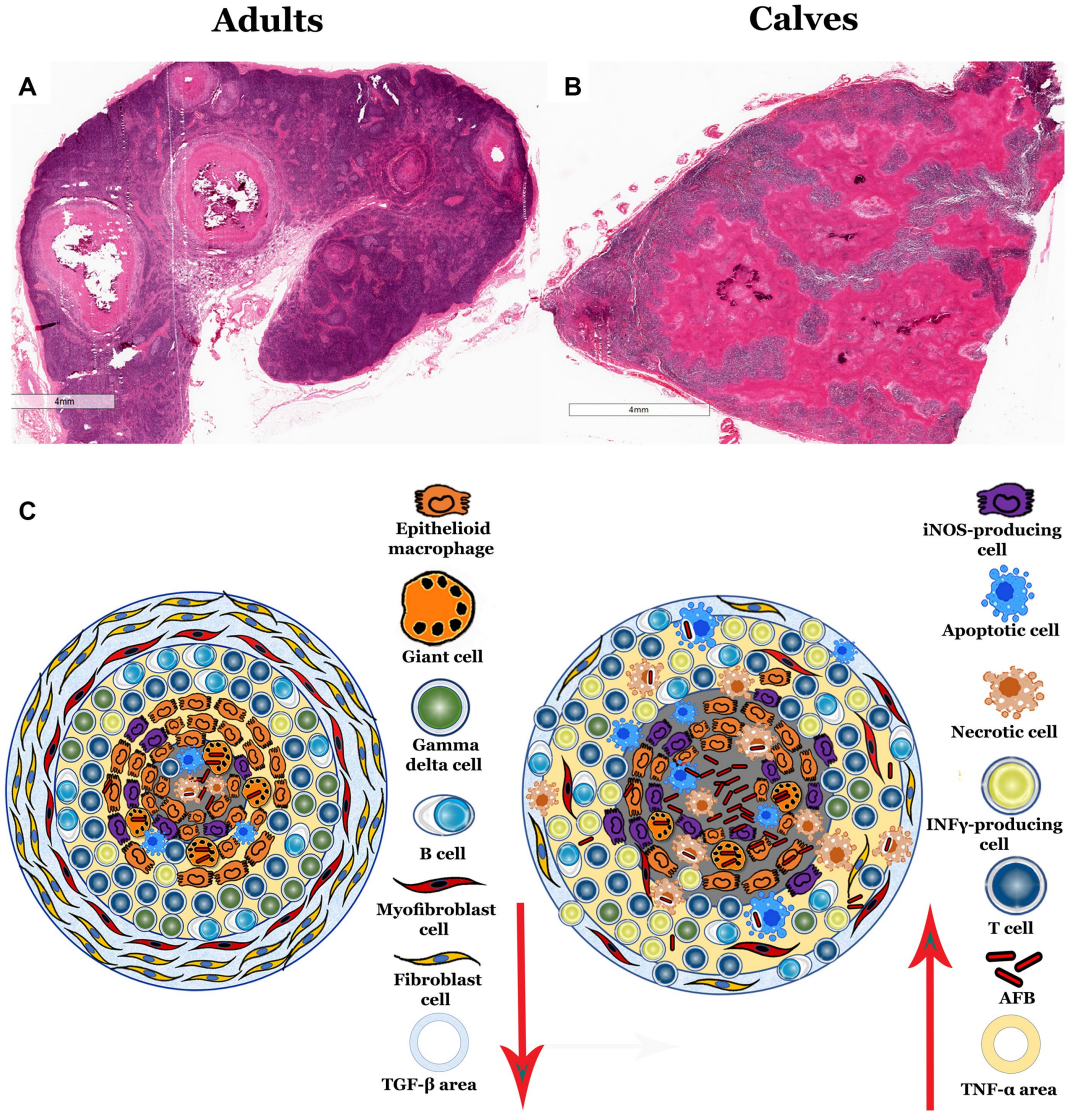
Propose schematic model of morphology and immune activity in granulomas from calves and adult cattle naturally infected with *Mycobacterium bovis*. **(A,B)**, 5× H&E staining of lymph node sections with infection-induced granulomas. **(A)** Adult bovine lymph node with different stage II and IV granulomas found in the lymph node medulla and cortex with the classic structure. **(B)** Calf lymph node, showing granulomatous lesions with a large amount of necrosis and absence of the connective tissue capsule. **(C)** Diagram summarizing the differences identified in granulomas, showing fewer MGCs, epithelioid MΦs, γδ T cells, B cells, fibroblasts, TGF-β and more iNOS, necrosis, T cells, IFN-α + cells, TNF-α, and mycobacteria cells in granulomas from calves compared to adult cattle.

Our results evidenced differences in the histological structure, number of bacteria, and immune response in granulomas from calves and adult cattle. In summary, granulomas in calves have more bacteria, no connective tissue capsules associated with disorganized structure; as well as fewer fibroblasts, myofibroblasts, epithelioid MΦs, MGCs, γδ T cells, B cells, and TGF-β immunereactivity than adult cattle. Taken together, these data suggest an exacerbated proinflammatory process that is inefficient in the control of *M. bovis* infection in naturally infected cattle (Figure 6). In a previous study from our group, we observed histological differences in granuloma architecture, such as the absence of the connective tissue capsule, more necrosis, and a greater number of AFBs in the granulomas of calves compared to adult cattle (9). To better understand the immune response and the number of bacteria found in these lesions, we used IHC and digital pathology analysis. We confirmed higher mycobacterium immunolabeling in granulomas from calves, mainly in extracellular and necrotic areas and in the cytoplasm of epithelioid MΦs and MGCs.

Our observations differ from previous reports in cattle experimentally infected with virulent strains of *M. bovis,* where the number of AFBs is low and located in the cytoplasm of giant cells (4, 18). However, we found a similar result in granulomas from adult cattle, where positive immunolabeling was mainly found in the cytoplasm of epithelioid MΦs and MGCs. In some lesions, it was impossible to detect positive staining, especially in stage III and IV granulomas. In monkeys, these types of lesions are capable of sterilizing mycobacteria in latent infections (19).

One important feature of this study was the use of polyclonal antibodies in the IHC since the protocol not only stained bacilli but also cellular remains. These remains were observed as vacuoles and cytoplasmic dust, possibly associated with cell debris due to mycobacteria processing and phagocytosis. Interestingly, both groups also showed mycobacteria immunolabeling in cells outside the granuloma. This had already been noted in previous studies, suggesting that mycobacteria are present outside the lesion, probably as the remains of phagocytosed bacteria (20–22).

The presence of a fibrotic capsule around the granuloma is a hallmark of bovine tuberculosis. The capsule is mainly composed of type I collagen, produced by fibroblasts and myofibroblasts. Using Masson’s trichrome staining, we previously observed fibrosis in granulomas from adult cattle, in agreement with previous reports (8, 23). However, the capsule was absent in the calf granulomas. To confirm whether calf lesions lacked fibroblasts, we performed IHCs of vimentin and α-SMA; as expected, immunolabeling was detected in fibroblasts and myofibroblasts forming the surrounding fibrous tissue capsule in late granulomas and intercalated in the cellular area in stage I and II granulomas of adult cattle. Surprisingly, vimentin and α-SMA immunolabeling of early-stage granulomas from calves was similar to that of adult cattle. Nevertheless, γδ T^+^ cells were d lesions with necrosis and calcification showed disorganized fibroblasts and myofibroblasts that did not form a capsule around the lesion. The amount of fibrosis correlated with the presence of TGF-β in granulomas and the differentiation of fibroblasts into collagen-producing myofibroblasts (24, 25). Using IHC, we observed less TGF-β in the granulomas of calves compared to those of adult cattle. This cytokine has been associated with the development of fibrosis in granulomas of cattle infected by *M. bovis* (8). The function of fibrotic capsules in the pathogenesis of tuberculosis and their formation around granulomas are incompletely understood, but some studies have associated them with the chronicity of the lesion, better control of bacteria, limitation of tissue damage, and latent infection (19, 25–27). In bovine tuberculosis, the fibrotic capsule is characteristic of stage III-IV granulomas and is composed mainly of type I collagen produced by fibroblasts. In naturally infected cattle, the thickness of the connective tissue capsule has been associated with fewer bacteria in granulomas (25). The absence of capsules in granulomas from calves with high bacterial burden suggests that capsules protect cattle naturally infected with *M. bovis*, but the factors that determine the formation and deposition of fibrous tissue in the external part of the lesions are still unclear. Interestingly, recent studies suggest that the fibroblasts forming a connective tissue capsule may be MΦs that undergo macrophage-myofibroblast transition (28). Our IHC results showed that MAC387, a protein found in MΦs and monocytes, was present in the cytoplasm and membrane of fibroblasts that formed the connective tissue capsule. This observation suggests the possibility of macrophage-myofibroblast transition in cattle granulomas.

Granulomas with higher bacterial burdens and no peripheral fibroblasts have been found in active tuberculosis infections in monkeys and humans associated with more proinflammatory cytokines (6, 19). In this study, higher immunolabeling of IFN-γ, TNF-α, and iNOS with fewer TGF-β was observed in granulomas from calves compared with adult cattle, suggesting a greater proinflammatory response. The high amount of IFN-γ in the calf granulomas suggests abundant CD3^+^ T cells in response to mycobacterial antigens. Strong whole-blood IFN-γ responses have been reported in calves as early as 1 month after *M. bovis* infection, and TNF-α production has been shown in BCG-vaccinated calves (29, 30). These two cytokines are essential for activating antimycobacterial mechanisms and inducing reactive nitrogen intermediates by activated MΦs, which play a crucial role in the intracellular killing of mycobacteria. However, despite higher iNOS production in granulomas from calves, they presented fewer epithelioid MΦs and MGCs compared to adult cattle. The cytotoxic activity induced by high iNOS concentrations might explain this contradictory result (31). Another possibility is that MΦs present in calf lesions were mainly derived from circulating blood. These MΦs are more proinflammatory and short-lived, and they depend more on glycolysis to produce energy than resident tissue MΦs (32). This finding is consistent with the idea that the MAC387 antibody detects an epitope on the calcium-binding protein MRP14 found in monocytes/MΦs that have recently infiltrated acutely inflamed tissues (33, 34). Similarly, we identified more MAC387^+^ cells in the uninjured tissue surrounding lymph node granulomas of calves compared to adult cattle, suggesting that the MΦs and monocytes detected in calves are mostly from the bloodstream and, therefore, more proinflammatory. We demonstrated that granulomas from calves present a more proinflammatory response than those of adult cattle; this type of microenvironment is associated with less TGF-β, possibly resulting in the lack of connective tissue observed. Granulomas are dynamic, spatially organized structures with a proinflammatory center that may present necrosis and a periphery of cells with a proinflammatory profile. From this study, we can infer that the granulomas of young bovines infected with *M. bovis* have a reduced anti-inflammatory response, which is why they lack adequate encapsulation of fibrous tissue.

The role of B cells and γδ T cells in the pathogenesis of bovine tuberculosis is incompletely understood. However, the presence of B cells in granulomas has been associated with better control of the infection since they are numerous in the granulomas with fewer bacteria (23). In this study, CD79^+^ lymphocytes were observed among the rest of the cells and around the lesion in different granuloma stages. Multifocal aggregates of CD79^+^ cells were also observed in some late stages. Finally, more CD79^+^ cells were found in the granulomas of adult cattle compared with those of calves (which had the highest bacterial burden). This result agrees with the finding that granulomas with more B lymphocytes tend to have fewer mycobacteria. Conversely, γδ T cells play a critical role in connecting innate and adaptive immunity in response to *M. bovis.* In peripheral blood, γδ T cells represent up to 70% of the lymphocytes in young animals and decline to an average of 10–20% in adult bovines (35). Moreover, WC1+ γδ T-cell from neonatal calves express high levels of INF-γ in response to IL-12 and IL-18 compared with adult animals (36). The higher percentage of T cells in calves suggests that they have an important participation in the immune system. In granulomas, γδ T cells are the first to arrive at the infection sites; they have been observed as early as 7–15 days after experimental infection with *M. bovis,* suggesting that they play a role in granuloma formation. Likewise, it has been reported that the number of γδ T cells is positively correlated with the stage of the granuloma, which agrees with our observations that the number of γδ T cells was higher in the granulomas of adult bovines, it is interesting to note that the highest concentration of γδ T cells is observed in stage IV. Although adult granulomas showed a higher concentration of γδ T cells, this does not correlate with the expression of INF-γ observed in these lesions (37, 38). Surprisingly, although these cells are increased in the circulation of calves, we found a small number in the granulomas. A possible explanation for this result is that γδ T cells in young cattle remain mainly in the bloodstream and are less present in the interstitium. These observations highlight the involvement of γδ T cells in the pathogenesis of tuberculosis in young animals.

One limitation of this study is that several factors that could affect the type of lesion remain unknown, including the grade of bacterial virulence, route of infection, bacterial dose, and date of infection. It is widely recognized that the pathology of bovine tuberculosis is multifactorial. However, this study emphasizes age as an important factor in the type of immune response and granuloma formation in cattle naturally infected with *M. bovis*. Granulomas from calves displayed a greater number of bacteria, lacked the connective tissue capsule, were associated with fewer and more disorganized fibroblasts and myofibroblasts, showed a predominance of proinflammatory cytokines (IFN-γ, TNF-α, and iNOS), and had fewer epithelioid MΦs, MGCs, γδ T cells, B lymphocytes, and TGF-β, compared to the granulomas from adult cattle. Our results suggest that calves have active-like tuberculosis with an exacerbated proinflammatory response that may be associated with more necrosis and a lower microbicidal capacity, making them more permissive to infection and dissemination of mycobacteria. This study highlights the importance of understanding the immune response and pathogenesis of bovine tuberculosis in young animals.

## Data availability statement

The original contributions presented in the study are included in the article/Supplementary material, further inquiries can be directed to the corresponding author.

## Ethics statement

The animal study was reviewed and approved by Ethics and Animal Welfare Committee of the Facultad de Medicina Veterinaria y Zootecnia, Universidad Nacional Autónoma de México (CICUA, FMVZ-UNAM), and complied with the Mexican guidelines for animal research (JAGP-074).

## Author contributions

JC-U and JAG-P conceived the experiments and wrote the original draft. JAG-P provided resources, project administration, and funding acquisition. JC-U and MAB-A collected and prepared samples. RH-P and CL-M advised on field data acquisition and analysis and provided scientific guidance during the experiment and drafting of the manuscript. JC-U, MJ-R, and GB-G performed the experiments and analyzed the data. SH-Y performed validation, writing-review, and editing. All authors contributed to the article and approved the submitted version.

## Funding

This study was financially supported by the Project of Programa de Apoyo a Proyectos de Investigación e Innovación Tecnológica (PAPIIT) IG201521 of the Universidad Nacional Autónoma de México.

## Conflict of interest

The authors declare that the research was conducted in the absence of any commercial or financial relationships that could be construed as a potential conflict of interest.

## Publisher’s note

All claims expressed in this article are solely those of the authors and do not necessarily represent those of their affiliated organizations, or those of the publisher, the editors and the reviewers. Any product that may be evaluated in this article, or claim that may be made by its manufacturer, is not guaranteed or endorsed by the publisher.
