# Supplementary material for: Mycobacterium bovis naturally infected calves present a higher bacterial load and proinflammatory response than adult cattle
Source: Front Vet Sci. 2023 Apr 27;10:1105716. doi: 10.3389/fvets.2023.1105716 (PMC10172680; doi:10.3389/fvets.2023.1105716)
Supplement: Supplementary file 1 [file Data_Sheet_1.docx]

Supplementary Material

# Supplementary Figures and Tables

| **Group** | **Case number** | **Age** | **Sex** | **Cause of death** |  |
| --- | --- | --- | --- | --- | --- |
|  |  |  |  |  |  |
| **Adults** | 1 | 4 Years | F | Infertility |  |
|  | 2 | 3 Years | F | Feedlot bloat |  |
|  | 3 | 3 Years | F | Traumatic pericarditis |  |
|  | 4 | 3 Years | F | Tibial fracture |  |
|  | 5 | 5 Years | F | Feedlot bloat |  |
|  | 6 | 5 Years | F | Infertility |  |
|  | 7 | 3 Years | F | Unknown |  |
|  | 8 | 5 Years | F | Feedlot bloat |  |
|  | 9 | 3 Years | F | Infertility |  |
|  | 10 | 2 Years | F | Traumatic pericarditis |  |
|  | 11 | 5 Years | F | Unknown |  |
|  | 12 | 5 Years | F | Feedlot bloat |  |
|  | 13 | 3 Years | F | Traumatic pericarditis |  |
|  | 14 | 5 Years | F | Unknown |  |
|  | 15 | 1 Year | F | Chronic feedlot bloat |  |
| **Calves** | 16 | 4 Months | M | Pneumonia |  |
|  | 17 | 8 Days | F | Cardio-respiratory failure. |  |
|  | 18 | 1 Month | F | Diarrhea and dehydration |  |
|  | 19 | 1.5 Months | F | Pneumonia |  |
|  | 20 | 3 Months | F | Pneumonia |  |
|  | 21 | 3 Months | F | Feedlot bloat |  |
|  | 22 | 2 Months | F | Peritonitis |  |
|  | 23 | 1 Month | F | Acute respiratory failure |  |
|  | 24 | 2.5 Months | F | Pneumonia |  |
|  | 25 | 3.5 Months | F | Acute respiratory failure |  |

F, female and M, male

**Supplementary table 1:** Causes of death in studied cattle.

**Antibodies used in immunohistochemistry and number of granulomas analyzed.**

| **Immunolabeling** | **Granulomas analyzed** | |
| --- | --- | --- |
|  | **Adults** | **Calves** |
| MAC387 | 72 | 146 |
| CD3 | 105 | 155 |
| CD79 | 103 | 215 |
| WC1 | 78 | 202 |
| α-SMA | 88 | 229 |
| Vimentina | 106 | 226 |
| TNF-α | 96 | 210 |
| INF-γ | 108 | 224 |
| TGF-β | 114 | 201 |
| iNOS | 116 | 254 |
| Anti-mycobacterium | 91 | 300 |
| **Total** | **1077** | **2362** |

**Supplementary table 2:** Antibodies used, and number of granulomas analyzed in each group.

## Supplementary Figures

**Supplementary Figure 1: Scheme summarizing experimental procedures:** Lymph nodes of Holstein-Friesian dairy cattle *M. bovis* naturally infected were included [1], the tissues were embedded in paraffin blocks [2], then serial sections of 4-5 µm were cut and immunolabeled [3] then tissue sections were scanned [4], and selected images were analyzed using the ImageScope software system, finally the number of positive pixels of each immunostaining was quantified [5-7]. (Created with [BioRender.com](https://biorender.com/), accessed on Sep 4, 2022)

**Supplementary Figure 2: Stages granulomas of calves *M. bovis* naturally infected show higher mycobacteria immunolabeling compared to adult cattle.**

**Supplementary Figure 3:** **Stage granulomas of calves naturally infected by *M. bovis* are associated with a lower number of fibroblasts and myofibroblasts. A** and **B**) Expression of vimentin and α-SMA immunolabeling was quantified in stages granulomas of adults and young cattle, Mann-Whitney test * * * *P*< 0.001 and * * *P*< 0.01 respectively.

**Supplementary Figure 4: Cell population differs between stages in granulomas from calves and adult cattle naturally infected with *M. bovis***. Average expression of immunolabeling in stage granulomas for MAC387 (macrophages), WC1 (γδ T cells), CD79 (B cells), and CD3 (T cells) respectively, from adult cattle and calves. Mann-Whitney test * *P*< 0.05, * * *P*< 0.01, and * * * *P*< 0.001. ND, not determined (because of lack to adult granulomas stage II).

**Supplementary Figure 5: Stages granulomas from calves show a more proinflammatory response than adults. A-D)**Average expression of immunolabeling for IFN-γ, the inducible form of nitric oxide synthase (iNOS), TNF-α, and TGF-β, respectively, in stages granulomas from adults and calves; Mann-Whitney test * *P*< 0.05, * * *P*< 0.01, and * * * *P*< 0.001.
