# Supplementary figures and images for: Mycobacterium bovis naturally infected calves present a higher bacterial load and proinflammatory response than adult cattle
Source: Front Vet Sci. 2023 Apr 27;10:1105716. doi: 10.3389/fvets.2023.1105716 (PMC10172680; doi:10.3389/fvets.2023.1105716)

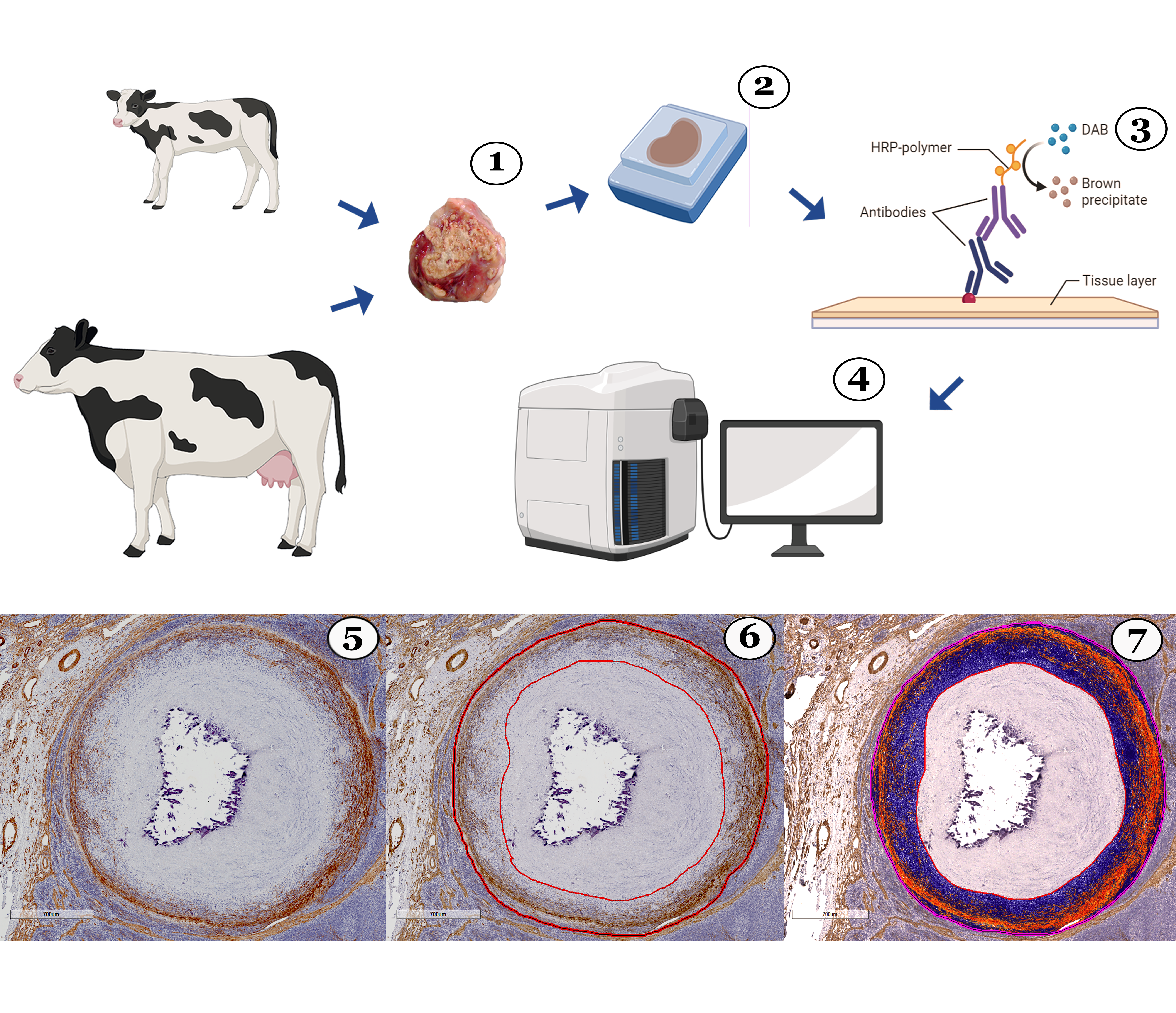

Supplement: Supplementary file 2 [file Image_1.TIF]

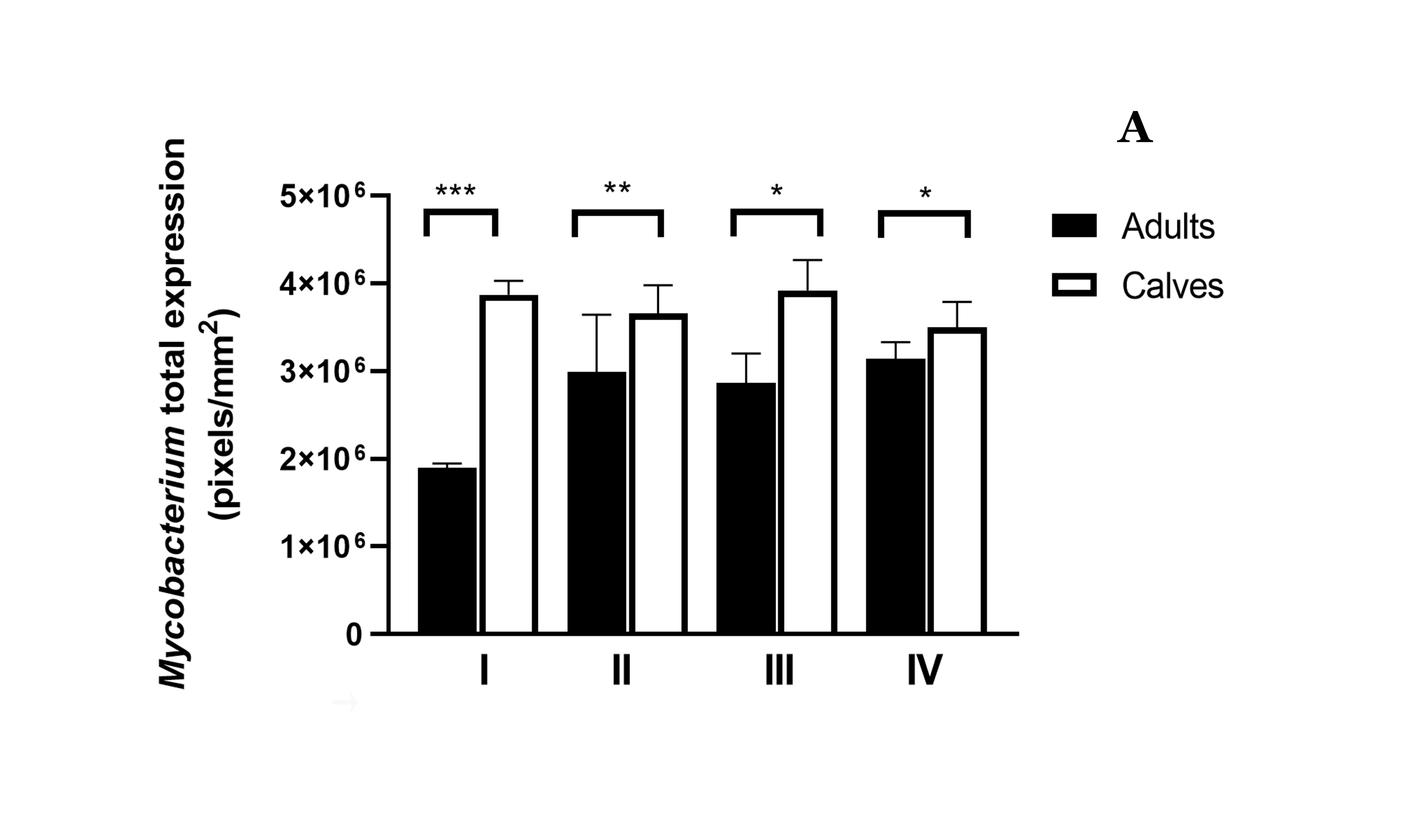

Supplement: Supplementary file 3 [file Image_2.TIF]

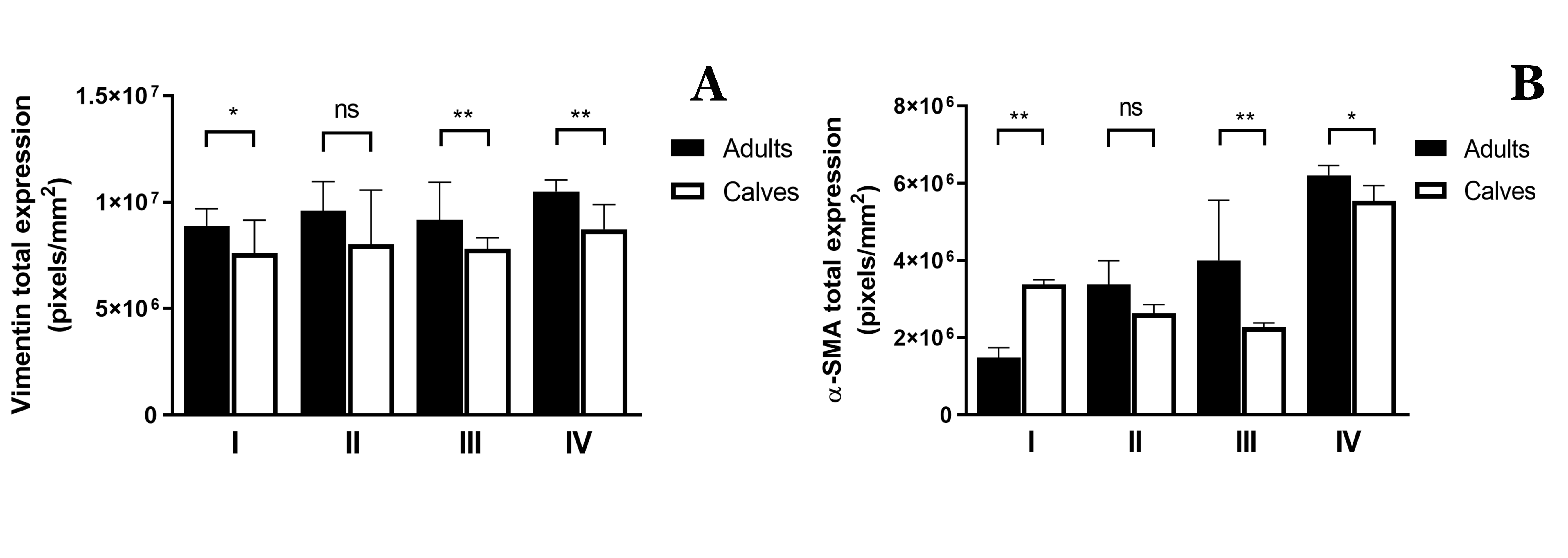

Supplement: Supplementary file 4 [file Image_3.TIF]

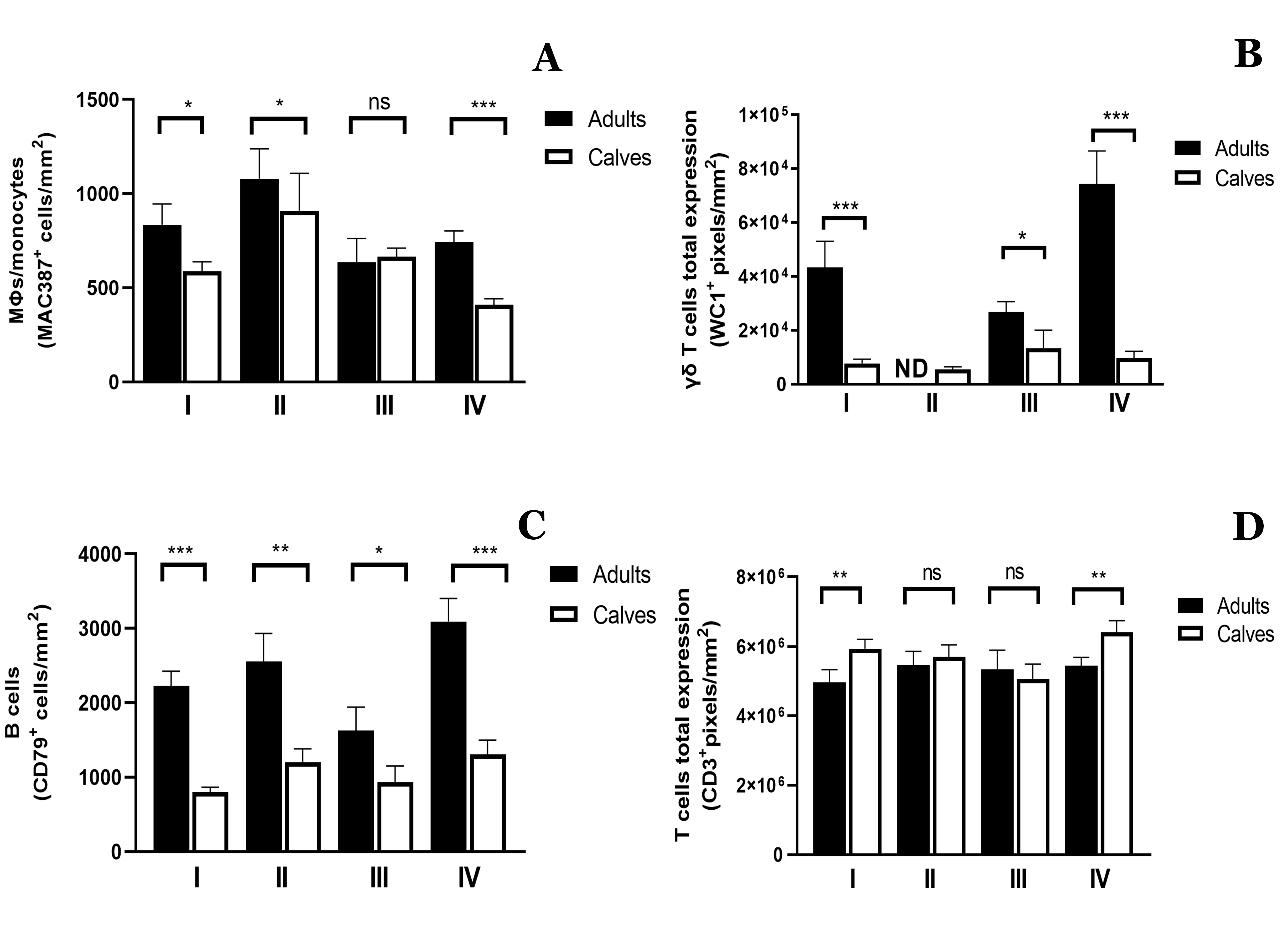

Supplement: Supplementary file 5 [file Image_4.TIF]

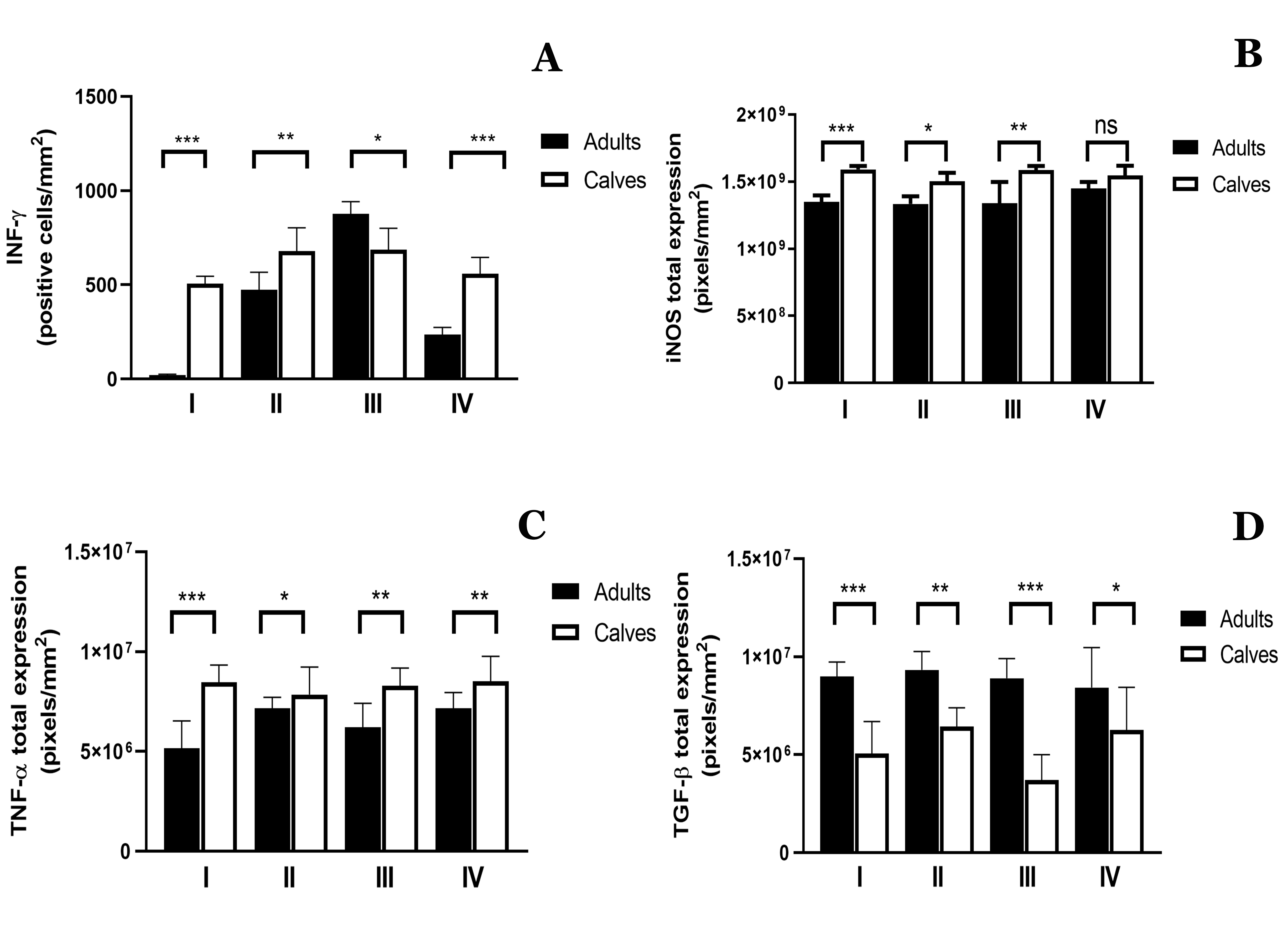

Supplement: Supplementary file 6 [file Image_5.TIF]
